# Pollution and Potential Ecological Risk Evaluation Associated with Toxic Metals in an Impacted Mangrove Swamp in Niger Delta, Nigeria

**DOI:** 10.3390/toxics11010006

**Published:** 2022-12-21

**Authors:** Davies Ibienebo Chris, Brilliance Onyinyechi Anyanwu

**Affiliations:** 1 Department of Fisheries, Faculty of Agriculture, University of Port Harcourt, Port Harcourt P.M.B. 5323, Rivers State, Nigeria; 2 Department of Environmental Health Sciences, College of Health Sciences, University of Sharjah, Sharjah P.O. Box 27272, United Arab Emirates

**Keywords:** mangrove, pollution, swamp, toxic metals, Niger Delta, Isaka–Bundu, ecological risk

## Abstract

Anthropogenic activities along coastal areas have contributed to the unwarranted discharge of toxic metals into mangrove swamps, posing risks to marine deposits and ecological environments. In this research, we studied the Isaka–Bundu tidal swamp area in the Niger Delta, which is an impacted mangrove creek located along the Bonny river, exposed to pollution pressures. The ecological risks (Er) of toxic metals in the sediments and water of the Isaka–Bundu tidal mangrove swamp followed a decreasing order (Cu > Zn > Cd > Cu > Pb > As), according to our results, while the potential ecological risk index (PERI) of the toxic metals in the sediments and water of the Isaka–Bundu tidal mangrove swamp can be said to have a very high ecological risk (PERI ≥ 600). The sediment pollution load index (PLI) was higher than 1 in all three analyzed stations, suggesting extremely toxic pollution. The enrichment evaluation shows that the studied stations have a moderate potential ecological risk of Cd, with the enrichment value for Pb showing low potential ecological risk. Our study shows that the Isaka–Bundu tidal mangrove swamp has a significant level of toxic metal pollution, which is evidence of the illegal activities performed in the Niger Delta.

## 1. Introduction

In recent years, there has been an increase in anxiety about the contamination of both fresh and brackish water in the Niger Delta, especially due to the threat associated with industrial and socioeconomic development, and this has contributed to increasing attention in the region [1,2,3]. Toxic metals arising from these anthropogenic activities have increased the discharges of different effluences into rivers and accumulated in sediments and crops, negatively impacting the ecology of river systems and having long-term effects on humans and other aquatic lives [4,5]. The sediment has been reported to be a sink of too many contaminants and pollutants, which may affect the surface water adversely [6,7].

Metals are the most persistent pollutants related to recent anthropogenic activities, and their toxicity varies depending on parameters, such as age, gender, and other individual variability [8]. Simply put, they have the potential to bioaccumulate in the food chain and endanger human health. Furthermore, because undernutrition is a leading cause of mortality in sub-Saharan Africa, ensuring food security for the continent’s natural deposit is critical, and a periodic assessment of metals’ dietary intake and associated dangers is required to determine the long-term risk to public health.

In recent times, high levels of toxic metals have been detected as a result of discharges from domestic, industrial, and other man-made activities in the Niger Delta [9,10,11,12]. A major ecological problem can be caused by toxic metals, one of the most harmful environmental pollutants [13,14,15,16]. The detrimental effects of trace metal pollution on fish cannot be ignored [17,18,19], because fishes are good indicators of trace metal pollution [20]. Studies have shown that toxic metals are important pollution intermediates in aquatic environments and public health [21,22]. They can induce certain diseases and be deposited on sediments and later immobilized [23,24].

In the absence of biodegradation, toxic metals accumulate throughout the food chain [25,26]. These accumulations in the aquatic environment and toxic metal contamination loads are affected by different environmental factors, which include human activities [27,28,29]. The rise in industrial activities and population in the urban regions have led to high concentration levels of toxic metals and organic pollutants in the aquatic environment [30,31], although the analyses of toxic metals in sediments have been used widely as a means of monitoring the pollution level in an ecosystem [32,33,34]. Some researchers have studied the different channels through which metals are transmitted from sediment to fish and from fish to humans [35,36,37]. Sediment may contain trace amounts of toxic metals, but when these metals accumulate to toxic levels under certain environmental conditions, such as anthropogenic activities, erosion, and natural weathering, they can harm the environment [13,38]. These toxic metals may represent a health concern to people if they bio-accumulate in fish through the food chain [11,39,40,41,42]. In aquatic ecosystems, sediment plays a vital role not only as a sink for pollutants but also as a component of the ecosystem as a whole [7,43], and several aquatic plants and animals rely on it for habitat, food, spawning grounds, and rearing grounds [44].

The crab is a part of the aquatic ecosystem that is consumed as food in many countries. A variety of minerals and high-quality proteins are found in seafood, including crustaceans and shellfish [45,46]. In addition, seafood is generally considered a very important part of a healthy, safe, and nutritious food, rich in minerals (e.g., Iron, Selenium, Zinc, Calcium, Iodine, and Copper), vitamins, fats, and high protein [47,48]. In many countries, crabs are an essential part of the aquatic ecosystem and are consumed as essential food for health and development. Crabs are also high in omega-3 polyunsaturated fatty acids, as well as other necessary elements, such as protein, carbs, ash, and energy [49,50,51]. As a result of the high cost of poultry, beef, and fish in developing countries, the less privileged are unable to eat them. Less privileged people can, however, obtain protein from less expensive animals, such as crabs [52,53]. The swimming blue crab (*Callinectes amnicola*), for example, is recommended for a healthy diet [54,55]. Several health benefits have been associated with seafood fatty acids, including chemopreventive effects on cancer [56,57]. It is, therefore, important to investigate the ecological implication of pollution and how to improve the environmental conditions.

This research examined the effects of hazardous metal pollution on swimming blue crabs (*Callinectes amnicola*) in the Isaka–Bundu mangrove swamp by estimating the environmental risk of the selected toxic metals in sediment, crab, and water. Consequently, the study will establish the level and concentration of these toxic metals in crabs, water, and sediments, which is adjacent to this populated region. This can be used to examine and report anthropogenic impacts on the ecosystems and assist in managing the risks posed by waste discharges.

## 2. Materials and Methods

### 2.1. Study Area

**Figure 1:**
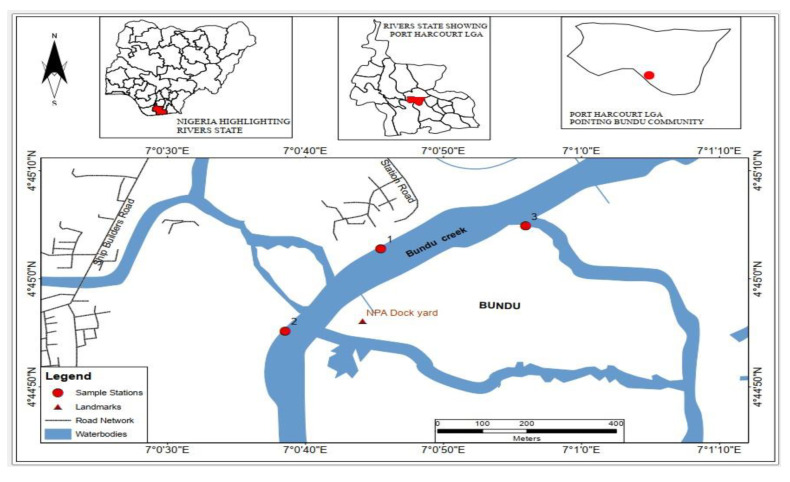
Section of the Isaka–Bundu Creek sampled and studied.

Isaka–Bundu tidal swamp is an impacted mangrove creek located along the upper reaches of the Bonny estuary exposed to pollution pressures that are emanated from anthropogenic activities and natural sources. This contaminated mangrove swamp is in Rivers State, which is an oil-rich state in the Niger Delta of Nigeria [11]. Three sampled stations were selected for the study: Station 1 (Isaka) is characterized by a substantial boom in industrial and agricultural activities, with increasing population growth. It is located near a heap of mudflat sediment, more like a dump site marred with oil sheen, and presumably formed by the tidal current dumping litter and other particulate contaminants with an abandoned bunkering site within view of this sampling point. Station 2 (Bundu-Ama) is a contact point with visible polluted sites, which are well spaced apart and close to a densely populated settlement, lining the tidal-swept mangrove swamp. This area is constantly bombarded with human, animal, and domestic wastes and runoffs and is visible on the shorelines. Station 3 (Creek Road/Dockyard) is at the Dockyard and also close to the Creek Road major market. The activities associated with this area generate wastes ranging from maintenance fluid discharge from the Dockyard, sewage, refuse, and other loads of commercial wastes from the market, conveniently dumped into the tidal river. These sampled stations are shown in Figure 1.

The sampled station 1 is located at Latitude 04°45′03.05″ N, Longitude 007°00′45.51″ E, station 2 lies at Latitude 04°44′55.35″ N, Longitude 007°00′38.40″ E, and station 3 is at Latitude 04°45′03.58″ N, Longitude 007°00′57.75″ E. Three sample stations were established along the Isaka–Bundu creek, after a reconnaissance survey of the areas. These communities experience impact from large quantities of effluents due to the activities that include improper domestic waste, artisanal crude oil discharges, and industrial waste from companies that has created a poor sanitary condition for the dwellers. These anthropogenic activities may have negatively impacted the vegetation with dead roots of mangroves and the surface of the water filled with spilt oil film [10].

### 2.2. Sample Collection

#### 2.2.1. Blue Crabs (*Callinectes amnicola*)

The blue crab samples were collected from the creeks of the Isaka–Bundu mangrove swamp. The swimming blue crabs (*Callinectes amnicola*) were caught from the river using a handheld net, baited with ox heart from each sampled station for the study, and the samples collected were preserved immediately in an icepack before being transported to the laboratory. Ten representative crabs were collected per station at each period of sampling with a total of one hundred and eighty representative crab samples collected within the sampling period. Crabs with a mean length of 12.10 ± 0.61 cm and weight of 119.6 ± 0.13 g were dissected. The soft tissues from 8 to 10 individual crabs were dissected, dried, and stored in a clean, clearly labelled, plastic container.

#### 2.2.2. Water Samples

Water samples were taken at the surface from the three sample sites using a clean 40 mL acid-washed, properly labelled, sterile polyethene screw-capped sample container. Before being sent to the laboratory, the water samples were acidified with 10 mL of 1:1 nitric acid: deionized water.

#### 2.2.3. Sediment Samples

An amount of 30 g of sediment samples was taken in a composite form from three separate sites at low tide once a month using an Ekman grab sampler and stored in a plastic bottle, previously treated with 10% nitric acid for 24 h and disinfected with de-ionized water. It was freeze-dried and the homogenized sediment particles were filtrated through a (0.071 mm) plastic mesh sieve and weighed before being taken to the laboratory [58]. These were done within 24 h after collection until analysis. A total of 36 sediment, water, and blue crab samples were collected once every month within the six months sampling period. Sampling was conducted between June 2021 and December 2021.

### 2.3. Sample Preparation and Digestion

Water samples were filtered using Whatman No. 1 filter paper and kept at 0 to 40 °C. To remove shell pieces from sediment samples, they were freeze-dried and put through a 1 mm transparent plastic filter. Sieved sediments were pulverized in an agate mortar. The powdered sediments were then placed on a clean nylon membrane screen (0.071 mm) and agitated to achieve a fine uniform powder. For digestion, a sample of 500 to 1000 g of dry sediment material was weighed. Microwave digestion was performed in acid-cleaned MDS-81D Teflon containers holding 5 mL of ultra-pure nitric acid and 2 mL of ultra-pure, concentrated hydrofluoric acid (HF). To ensure homogeneity and process efficacy, each digestion batch includes at least one reagent blank, a representative reference standard, and a sample replication. At 2000 °C, the samples were digested for 30 to 40 min. The jars were opened after at least 1 h of cooling, and 0.9 g of boric acid was added to dissolve the fluoride precipitates. The containers were resealed and returned to the microwave digestion system for another 20 to 30 min. After at least 1 h of chilling, the digested sample was transferred to a graduated plastic test tube with an additional 0.5 mL HF and filled to capacity (either 15 or 50 mL) with Milli-Q water, using the accepted techniques for examining water and wastewater.

Crab tissue samples weighing 0.5, 0.01 g were measured directly into MDS-81D Teflon digestion containers. Each vessel received ten millilitres of ultra-pure nitric acid before being heated with an XT-9800 pre-treatment heater to 1000 °C until nearly all of the nitrogen dioxide was released. Prior to microwave digestion, a 4 mL aliquot of concentrated HNO3:HF (1:1 *v*/*v*) acid mixture was applied. Each digestion batch had a representative reference standard, a sample replication, and at least one reagent blank to ensure homogeneity and process effectiveness. Three phases made up microwave digestion: 0.5 MPa for one minute, 1.0 MPa for two minutes, and 1.5 MPa for three minutes. The digested sample was transferred to a graduated plastic test tube and the volume was brought up to 100 mL with Milli-Q water after waiting at least 1 h for chilling [9,36].

### 2.4. Quality Assurance

To generate an analytical curve, the apparatus was calibrated using buck-certified atomic absorption standards for a number of hazardous metals. To prevent equipment drift, a reagent blank was initially run at intervals for every 10 samples analyzed. Recovery rates varied between 82 and 110%. Metal concentrations were analyzed using atomic absorption spectrophotometry (Model 210 VGP, Buck Scientific, Norwalk, CT, USA) in soil and biota samples. Table 1 displays the procedure description and wavelength (nm). The mean values of each sample were run in duplicate and reported.

### 2.5. Sample Analysis

#### 2.5.1. The Pollution Load Index (PLI)

The amount by which the metal content in the sediment exceeds the background concentration is indicated by the pollution load index (PLI). It offers thorough details on the amount of metal pollution present in a specific sample [59]. The nth root of the concentrations’ multiplications is referred to as the pollution load index (PLI). A PLI score of more than 1 denotes pollution, whereas a number less than 1 denotes no pollution [60]. Tomlinson et al. [61] presented the following method for calculating PLI.

PLI = (CF1 × CF2 × CF3 ⋯ × CFn)1∕n(1)

where CF is the contamination factor and n is the number of metals studied (six in this case).

#### 2.5.2. Contamination Factor (CF)

This is a pollution indicator associated with single toxic metal [62]. The CF is expressed as the ratio between the content of each metal to the background value.

CF = C_metal_/C_background_ (2)

where C_metal_ is the mean metal content in the sample, C_background_ is the mean natural background value of the metal stipulated by the Department of Petroleum Resources (DPR). The toxic metal standards were used as baseline values. The ratio of the measured concentration to the natural abundance of a given metal had been proposed as the index. CF is classified into four grades for monitoring the pollution of a single metal over a period of time [63]: low degree (CF < 1), moderate degree (1 ≤ CF < 3), considerable degree (3 ≤ CF < 6), and very high degree (CF > 6).

#### 2.5.3. Ecological Risk Factor (ERi)

The risk factors analysis (ERi and PER) evaluates the ecological risk potential of a single contaminant and toxic effect, as well as the impacts of several metal contaminants in sediment or soil.

The equations for the parameters are:

ERi = (Tr) × (CF)(3)

where CF represents the contamination factor and Tr is the toxic response factor viz.: Cr = 2, Pb = Cu = 5, V = 2, Cd = 30, and Zn = 1, [62], with Ni = 5 [64,65] and Fe not available. Therefore, the final calculations were obtained by excluding the values of Fe. The values obtained were interpreted according to terms used to interpret ERi [61]. Based on Hakanson [62], five terms are used to categorize ecological threats. There are four categories: low potential ecological danger (<40), moderate potential ecological risk (40 < 80), significant potential ecological risk (80 < 160), high potential ecological risk (160 < 320), and extremely high potential ecological risk (≥320).

#### 2.5.4. Potential Ecological Risk Index (PERI)

To estimate the ecological risk level of toxins and toxic metals in the environment, the potential ecological risk index is utilized [65]. They could be harmful to the environment and its inhabitants [62]. The potential ecological risk index (PERI) was evaluated as the sum of ecological risk factors indexes (ERi) for specific metals in a sample [62].

The equation for the potential ecological risk index (PERI) is as follows:

PERI = ∑Eri1 + Eri2 + Eri3 + Eri4 + Eri5………Erin(4)

where n = the number of toxic metals and EF = single index of the ecological risk factor. The following expressions have been used by Hakanson [62] for the potential ecological risk index: <150 (low ecological risk), 150 < 300 (moderate ecological risk), 300 < 600 (considerable ecological risk), and ≥600 (very high ecological risk).

#### 2.5.5. Contamination Degree (CD)

This parameter is the total amount of contamination factors. It provides a clue as to how contaminated the sediments from a sampling location are overall. Hakanson [62] proposed that the classification CD < 6 is a low degree of contamination, 6 ≤ Cd < 12 is indicative of a moderate degree of contamination, 12 ≤ CD < 24 indicates a considerable degree of contamination, and CD ≥ 24 represents a very high degree of contamination.

#### 2.5.6. Geo-Accumulation Index (Igeo)

The geo accumulation index (Igeo) established by Muller [66] could be used to quantify the degree of contamination from toxic metals. The geo-accumulation index has been frequently used to analyze sediment pollution [67]. The index has seven grades, ranging from 0 to 6, with each grade having its own set of points (uncontaminated to extremely contaminated). Müller [66] suggested seven Igeo classifications, which are as follows: Igeo ≤ 0, uncontaminated (Class 0); 0 < Igeo ≤ 1, uncontaminated to moderately contaminated (Class 1); 1 < Igeo ≤ 2, moderately contaminated (Class 2); 2 < Igeo ≤ 3, moderately to heavily contaminated (Class 3); 3 < I geo ≤ 4, heavily contaminated (Class 4); 4 < Igeo ≤ 5, heavily to extremely contaminated (Class 5); and Igeo > 5, extremely contaminated (Class 6). It is calculated as:

(5) $$\mathrm{Igeo}=\log2\left(\frac{\mathrm{Cn}}{1.5\mathrm{Bn}}\right)$$

Cn denotes the average concentration of the toxic metal in the water analyzed samples. The reference value is Bn.

#### 2.5.7. Degree of Contamination (DC)

The degree of contamination provides details on the environmental hazards posed by sediment, owing to the presence of several trace metals. It was developed by Hakanson [62] and has been used by Essien et al. [68]. The equation is as follows:

DC = ∑Pb + ∑Cd + ∑Cu + ∑Hg + ∑Cr……∑n(6)

where CF1 denotes the metal contamination factor. DC greater than 24 is considered a very high degree of contamination while DC ≤ 6 is a low degree of contamination.

#### 2.5.8. Enrichment Factor (EF)

The enrichment factor (EF) analysis of the measured toxic metals was calculated using the equation by Buat-Menard and Chesselet [69].

EF = (Cn/Cref sample)/(Bn/Bref)(7)

where Cn is the concentration of metal detected in the sample, Cref is the concentration of the reference material (in this research, Fe), Bn is the concentration of the studied metal in the background, and Bref is the concentration of the reference element (Fe). Enrichment factor classes were predicted based on the following categories: EF < 2 absence to insignificant enrichment, EF = 2–5 fair enrichment, EF = 5–20 severe enrichment, EF = 20–40 severe enrichment, and EF > 40 exceptionally high enrichment [70,71].

### 2.6. Statistical Analysis

The changes in hazardous metal concentrations in sediment and benthic fauna between the wet and dry seasons were determined using a one-way analysis of variance (ANOVA) at a significant threshold of 0.05, and there were calculated standard errors. IBM SPSS Statistics 20 and Microsoft Excel 2010 were used to execute all computerized statistical analyses.

## 3. Results

### 3.1. Concentrations of the Toxic Metals

Table 2 shows the mean concentrations of the toxic metals (Cd, Pb, Zn, Fe, As, and Cu) collected from the different mediums in the Isaka–Bundu tidal mangrove swamp. The results were represented as concentrations in mg kg^−1^ dry weight for fish and sediment, and in mg/L for water.

### 3.2. Contamination Factor (CF)

The contamination factor (CF) also used to assess the samples contamination was estimated for individual toxic metals in the studied sediment, water and *C. amnicola* samples, and the results are presented in Table 3. The results showed that the mean CF values of the toxic metals in the water decreased in similar trends for Isaka, Bundu-Ama, and Dockyard in the following order: Cu > Cd > Zn > Fe > Pb > As. The mean contamination factor values of the toxic metals in *C. amnicola* decreased in the following order: Zn > Cd > Fe > Cu > Pb > As in Isaka, Zn > Cu > Cd > Fe > As > Pb in Bundu-Ama, and Zn > Cd > Cu > As > Fe > Pb in Dockyard while the mean contamination factor values of the toxic metals in the water for Isaka, Bundu-Ama, and Dockyard followed this order: Cd > Cu > Zn > Fe > Pb > As, Cd > Zn > Cu > Pb > Fe > As, and Cd > Cu > Zn > Pb > Fe > As, respectively.

As indicated in Table 3, in Isaka, the degree of contamination (DC) varied from 108.52 to 2306.1, 103.97 to 4447.0 in Bundu-Ama, and for Dockyard it was 120.79 to 3368.19. The degrees of contamination recorded in all sample stations are all greater than 24, thereby being considered very high [72]. However, the potential ecological risk index for *C. amnicola* at the station declined in the following order: Dockyard > Bundu-Ama > Isaka, based on the mean values of the degree of contamination. The sediment samples from the three sample sites were collected in the following order: Bundu-Ama > Dockyard > Isaka. Water flowed in the following sequence: Dockyard > Bundu-Ama > Isaka. Across the station, the possible ecological risk index for the medium fell in the following order: sediment > water > *C. amnicola.*

### 3.3. Ecological Risk Assessment

**Figure 2:**
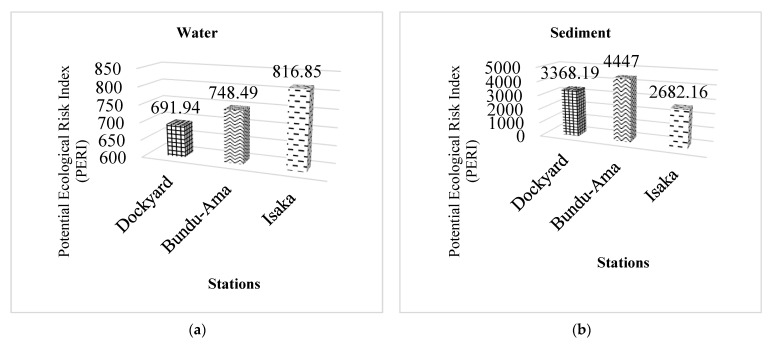
Heavy metals’ potential ecological risk index (PERI) in the Isaka–Bundu tidal mangrove swamp. (**a**) The PERI values recorded for water in all the stations; (**b**) The PERI values recorded for sediment in all the stations.

The prospective ecological risk index (PERI) is calculated using the ecological risk index (Er) of each toxic metal in the sediments. The potential ecological risk index (PERI) was used to quantify the ecological sensitivity of toxic metal pollution in the Isaka–Bundu tidal mangrove swamp, based on toxic metal toxicity and environmental reactions [64]. Figure 2 depicts the findings of the ecological risk factor (Er) and prospective ecological risk index (PERI) evaluations (a and b). The Er of toxic metals in the Isaka–Bundu tidal mangrove swamp sediments and water can be graded in the following decreasing order: Cu > Zn > Cd > Cu > Pb > As, as presented in Table 4.

### 3.4. Pollution Load Index (PLI)

**Figure 3:**
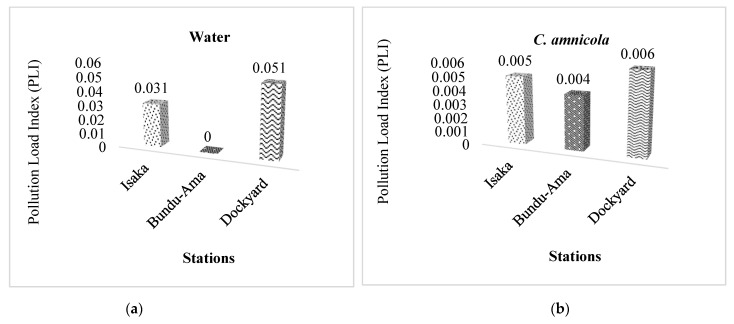

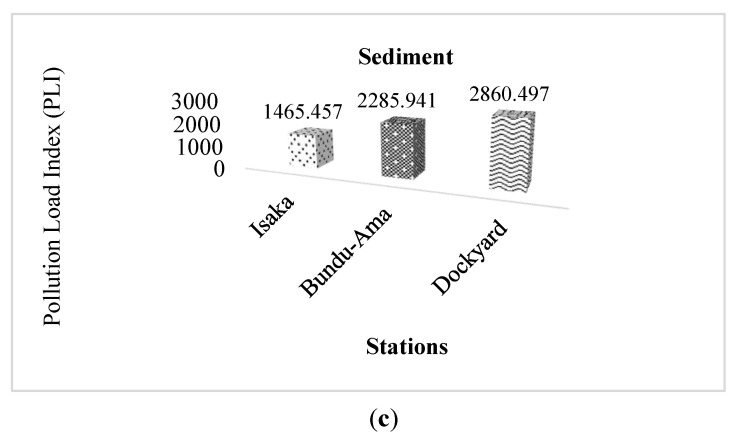
The pollution load index (PLI) in Isaka–Bundu tidal mangrove swamp. (**a**) The pollution load index (PLI) values for water; (**b**) The pollution load index (PLI) values for *C. amnicola*; (**c**) The pollution load index (PLI) values for sediment.

The calculated PLI was to identify the pollution level by integrating all analyzed toxic metals in three sample stations, including crab, water, and sediment. The results of PLI analysis support the same results recorded for the degree of contamination, which shows that the crab, water, and sediment are contaminated with Cd, Pb, Zn, Fe, As, and Cu in the Isaka–Bundu tidal mangrove swamp. This is presented in Figure 3a–c. The pollution load index calculated was lower than 1 in the water and crab analyzed, which suggests less anthropogenic loading in the two samples while the sediment PLI was higher than 1 in all three analyzed stations, which suggests extremely toxic pollution with anthropogenic loading, especially from the illegal refining waste around the study sites. There is an increased risk of rising levels of pollution of Cu > Zn > Cd > Cu > Pb > As in sediment in virtually all three study stations.

### 3.5. Geo-Accumulation Index (Igeo)

The geo-accumulation index (Igeo) of each metal element was calculated using the geo-accumulation evaluation formula by Muller (1969) [66]. The estimated geo-accumulation indices are regarded as the most accurate and widely used indicator for assessing toxic metal accumulations in the aquatic environment [73]. The Igeo was estimated using the element’s background geochemical value on an average scale [74]. Table 5 gives the generated Igeo values.

### 3.6. The Enrichment Factor

The enrichment factor for metals in sediment is shown in Table 6 from the three study locations. The Cd-enrichment examination suggests that the three stations (Isaka, Bundu-ama, and Dockyard) have a high potential ecological threat, but the Pb-enrichment value at the three stations has a low potential ecological risk.

## 4. Discussion

In this study, we investigated the effects of toxic metal pollution on swimming blue crabs in this study (*Callinectes amnicola*) in the Isaka–Bundu mangrove swamp by estimating the ecological risk of the selected toxic metals in the environment and the shell crab via examining the toxic metals in the sediment and water. The studies revealed that the mean CF values of toxic metals in water declined in similar trends for Isaka, Bundu-Ama, and Dockyard in the following order: Cu > Cd > Zn > Fe > Pb > As. There was a decrease in the contamination of the toxic metals in *C. amnicola*, which follows this order: Zn > Cd > Fe > Cu > Pb > As in Isaka, Zn > Cu > Cd > Fe > As > Pb in Bundu-Ama, and Zn > Cd > Cu > As > Fe > Pb in Dockyard. The toxic metal contamination in sediment in Isaka, Bundu-Ama, and Dockyard followed this order: Cd > Cu > Zn > Fe > Pb > As, Cd > Zn > Cu > Pb > Fe > As, and Cd > Cu > Zn > Pb > Fe > As; respectively. The water and *C. amnicola* in Isaka, Bundu-Ama, and Dockyard showed a low level of Cd pollution, with the exception of sediments, which had the highest value (CF > 6), which is regarded as a very high level of contamination [63]. This could be attributed to serious anthropogenic pollution from the artisanal crude oil refining waste discharged indiscriminately around the three sampled stations [72,75]. Fe and Pb showed low degree (CF < 1) contamination in the three mediums across the three stations. Zn showed a low degree of contamination in the water across the stations, whereas it recorded a considerable degree (3 ≤ CF < 6) of contamination in *C. amnicola* across all stations.

The highest CF value of 24.98 was recorded in the sediment at Bundu-Ama with a very high degree of contamination. Such a sample is considered to be very highly contaminated [76,77]. Cu contamination in the sediments was quite high (CF > 6) in all three sites; there was a moderate degree (1 ≤ CF < 3) of contamination in water and a low degree (CF < 1) of contamination in *C. amnicola.* These results also confirm that the artisanal refining and other associated anthropogenic activities along these study areas might have led to the pollution with hazardous metals since various pieces of research have already highlighted the impacts of industrial pollution in Niger [78,79,80,81,82].

Furthermore, the degrees of contamination recorded in all the sample stations were all greater than 24, which is considered very high. This could be indicating severe impact from anthropogenic activities and their associated waste pollutants [68]. This agrees with [83,84,85], who obtained similar results, indicating serious anthropogenic pollution. The Er of toxic metals in the Isaka–Bundu tidal mangrove swamp sediments and water was rated in the following order: Cu > Zn > Cd > Cu > Pb > As, whereas the potential ecological risk index (PERI) of toxic metals in the sediments and water of the Isaka–Bundu tidal mangrove swamp is very high (PERI ≥ 600). At three locations, the mean Er index values for Cd, Zn, Pb, and As were less than 40 (Er < 40; i.e., low potential ecological damage). Cu was a significant possible ecological danger 80 ≤ Er < 160) in the Bundu-Ama Sediment, whereas Cu and Cd were both significant potential ecological risks (80 < 160) in the Isaka, Bundu-Ama, and Dockyard sediment. Fan et al. [86] obtained a similar finding in the polluted soil of three mining regions in central China. Zn was a high potential ecological risk (160 < 320) in the sediment at all three stations, while Cu was a very high ecological risk (≥320). Similar observations were reported by Rao et al. [87] in inshore sediments of the Yellow River estuary. Peter et al. [88] observed a similar result, with these toxic metals in the sediment posing a high potential for ecological damage.

The sediment PLI was higher than 1 in all three analyzed stations, suggesting extremely toxic pollution. The enrichment evaluation shows that three stations (Isaka, Bundu-Ama and Dockyard) have a moderate potential ecological risk of Cd, while the enrichment value for Pb at the three stations shows low potential ecological risk. It was also observed that sediment and water at Isaka and Dockyard recorded low potential ecological risk and a moderate potential ecological risk in the sediment at Bundu-Ama. Our study observed that all three stations fell under the very low enrichment of As. The Igeo classes indicate variation in the quality of the sediment and content of the different sites and contamination with an order of Cd > Cu > Zn > Fe > Pb > As from moderately contaminated to highly contaminated. Our findings showed that Isaka, Bundu-Ama, and Dockyard recorded Igeo values of the metals (As, Fe, Cu, Zn, Pb and Cd) in water and *Callinectes amnicola* and As, Pb, Zn, and Fe in sediment, which fell in class 1 (0 < Igeo < 1), implying that they are moderately contaminated. Meanwhile, the Cd in the sediment in Isaka, Bundu-Ama, and Dockyard fell in class 6 (Igeo > 5), implying that they were extremely contaminated with Cd [66]. Cu in the sediment fell in class 2 (Igeo < 2) across the stations, showing that they were moderately contaminated, while Zn fell in class 6 (Igeo > 5) in Bundu-Ama showing that it is highly contaminated with Zn. These classes indicate variation in the quality of the sediment and content of the different sites and contamination with an order of Cd > Cu > Zn > Fe > Pb > As. The reference values might cause an evaluation disagreement. However, Duncan et al. [89] and Yahaya et al. [90] observed that Pb, Zn, Fe, and As were moderately polluted, Cd was severely contaminated, and Zn was highly contaminated, using the background level as a reference in one of the research locations. This shows that the three research locations were contaminated by Cd, despite the varying reference levels utilized.

The analysis of Cd enrichment suggests that three stations (Isaka, Bundu-Ama, and Dockyard) have a high potential ecological threat, but the enrichment value for Pb at the three stations suggests low potential ecological risk [70]. For Zn, sediment and water in Isaka and Dockyard indicated a low potential ecological threat, whereas sediment at Bundu-Ama had a moderate potential ecological risk. It was also discovered that all three stations had relatively low As enrichment. A really low enrichment level shows that activities around the three stations had no substantial influence on As and Pb accumulation in comparison to the natural background levels in the studied region. [91]. This gives a clear suggestion that the toxic metal was the major anthropogenic activity, leading to the accumulation of Cd, Zn, and Cu in the study areas. This agrees with Kwon et al. [92] who reported that the bioavailability and toxicity of the metals in the sediment samples may not only depend on the concentrations of metals but also on their existing chemical composition. According to Mohammed and Abdu [93], the enrichment of Cd, Zn, and Cu in the region might be attributed to human activities, which could include evident artisanal refining operations. This is to say that the illegal crude oil refining and processing may be the major factors that introduced Cd, but Zn and Cu were also introduced into the water body; therefore, they may be created through different processes, which can be chemical, physical, and biological [90,94,95].

## 5. Conclusions

In conclusion, this study reveals that the Isaka–Bundu tidal mangrove swamp has a high degree of hazardous metal contamination, which is visible in criminal operations in the Niger Delta. As a result, it is recommended that strong legislation and regulatory measures be enacted and implemented by legislators against unlawful industrial activity, particularly those next to this water body in the Niger Delta. Furthermore, the toxicity evaluations of Cd and Pb must be examined further in other Niger Delta creeks in order to determine their potential human implications on agricultural produce/seafood and to develop a strategy for mitigating these metal contaminants.

## Figures and Tables

**Table 1:** Toxic metal analysis procedure.

S/n	Elements	APHA Method	Wavelength (nm)	Method Description
1.	Cd	APHA 3111B	326.11	Direct Air–Acetylene Flame Method
2.	Pb	APHA 3111B	202.20	Direct Air–Acetylene Flame Method
3.	Zn	APHA 3111B	213.86	Direct Air–Acetylene Flame Method
4.	Fe	APHA 3111B	248.30	Direct Air–Acetylene Flame Method
5.	As	APHA 3111B	197.20	Direct Air–Argon Flame Method
6.	Cu	APHA 3111B	217.89	Direct Air–Acetylene Flame Method

**Table 2:** Mean concentrations (mg kg^−1^ dry weight for fish and sediment; mg/L for water) of Pb, Cd, Zn, Fe, As, and Cu from the various media in the Isaka–Bundu tidal mangrove swamp.

Source	Sampling Site	Station	Cd	Pb	Zn	Fe	As	Cu
Crab (mg kg^−1^)	Isaka	1	0.001 ± 0.000	0.004 ± 0.001	97.09 ± 0.001	10.73 ± 0.000	0.002 ± 0.001	0.69 ± 0.001
	Bundu-Ama	2	0.001 ± 0.000	0.002 ± 0.001	100.01 ± 0.001	12.67 ± 0.000	0.002 ± 0.001	0.78 ± 0.001
	Dockyard	3	0.001 ± 0.000	0.005 ± 0.003	117.34 ± 0.001	12.52 ± 0.000	0.002 ± 0.001	0.67 ± 0.001
Water (mg/L)	Isaka	1	0.05 ± 0.003	0.060 ± 0.003	8.29 ± 0.003	25.12 ± 0.003	0.001 ± 0.00	136.35 ± 0.01
	Bundu-Ama	2	0.05 ± 0.01	0.074 ± 0.001	9.84 ± 0.01	46.41 ± 0.00	0.000 ± 0.00	147.35 ± 0.01
	Dockyard	3	0.05 ± 0.001	0.094 ± 0.001	14.50 ± 0.07	39.20 ± 0.07	0.001 ± 0.00	160.05 ± 0.01
Sediment (mg kg^−1^)	Isaka	1	2.33 ± 0.001	6.160 ± 0.001	215.44 ± 0.00	1609 ± 0.67	0.012 ± 0.00	473.19 ± 0.0
	Bundu-Ama	2	3.84 ± 0.00	8.510 ± 0.00	209.02 ± 0.00	1634 ± 0.67	0.013 ± 0.00	531.03 ± 0.00
	Dockyard	3	2.59 ± 0.006	12.07 ± 0.003	246.41 ± 0.003	1846 ± 0.58	0.016 ± 0.003	596.73 ± 0.003
DPR *	Standard		0.8	85	140	38,000	1.0	36

* Department of Petroleum Resources (DPR).

**Table 3:** Contamination factor (C_f_) and degree of contamination (DC) of Cd, Pb, Zn, Fe, As, and Cu in the Isaka–Bundu tidal mangrove swamp.

	Isaka			Bundu-Ama			Dockyard		
Toxic Metals	Water	*C. amnicola*	Sediment	Water	*C. amnicola*	Sediment	Water	*C. amnicola*	Sediment
Cd	5.4 × 10^1^	1.0 × 10^2^	23.3	5.1 × 10^1^	1.0 × 10^2^	38.42	5.4 × 10^1^	1 × 10^2^	25.88
Pb	2.9 × 10³	2.0 × 10^4^	3.08 × 10^1^	3.7 × 10^5^	1.0 × 10^4^	4.25 × 10^1^	4.7 × 10^3^	2.5 × 10^5^	6.03 × 10^1^
Zn	1.27 × 10^1^	1.484	3.294159	1.504 × 10^1^	1.529189	24.984	2.22 × 10^1^	1.7942354	3.767798
Fe	6.5 × 10^3^	2.78 × 10^3^	4179 × 10^1^	1.21 × 10^2^	3.291 × 10^3^	4.2 × 10^1^	1.02 × 10^2^	3.25 × 10^5^	4.8 × 10^1^
As	7.69 × 10^5^	1.53 × 10^4^	9.23 × 10^4^	0.0	1.538 × 10^4^	1.0 × 10^3^	7.69 × 10^5^	1.5384 × 10^4^	1.23 × 10^3^
Cu	1.947	9.885 × 10^4^	6.759786	2.105	1.114 × 10^2^	7.5859	2.286414	9.6285 × 10^4^	8.524686
**DC**	169.868	108.52	2306.12	203.725	113.465	3811.37	213.902	130.543	2703.813

**Table 4:** Ecological risk factor and the potential ecological risk index (PERI) of the toxic metals in the Isaka–Bundu tidal mangrove swamp.

	Isaka		Bundu-Ama			Dockyard
Toxic Metals	Water	Sediment	Water	Sediment	Water	Sediment
Cd	1.62	69.90	1.53	115.26	1.62	77.64
Pb	0.29	30.78	0.37	42.55	0.47	60.34
Zn	8.29	215.44	9.84	1634.00	14.50	246.41
As	0.01	0.12	0.00	0.13	0.01	0.16
Cu	681.73	2365.93	736.75	2655.07	800.25	2983.64
PERI	691.94	2682.16	748.49	4447.0	816.85	3368.19

**Table 5:** Toxic metal geo-accumulation index (Igeo) and pollution load index (PLI) in the Isaka–Bundu tidal mangrove swamp.

	Isaka			Bundu-Ama			Dockyard		
Toxic Metals	Water	*C. amnicola*	Sediment	Water	*C. amnicola*	Sediment	Water	*C. amnicola*	Sediment
Cd	0.108	0.002	4.676	0.102	0.002	7.710	0.108	0.002	5.194
Pb	0.001	0.000	0.062	0.001	0.000	0.085	0.001	0.000	0.121
Zn	0.025	0.298	0.661	0.030	0.307	5.014	0.044	0.360	0.756
Fe	0.001	0.001	0.084	0.002	0.001	0.085	0.002	0.001	0.096
As	0.000	0.000	0.000	0.000	0.000	0.000	0.000	0.000	0.000
Cu	0.391	0.002	1.357	0.422	0.002	1.522	0.459	0.002	1.711

**Table 6:** Enrichment factor for toxic metals in the Isaka–Bundu tidal mangrove swamp.

	Isaka	Bundu-Ama	Dockyard
Toxic Metals	Sediment	Sediment	Sediment
Cd	52.89	87.22	58.75
Pb	0.70	0.97	1.37
Zn	7.48	56.72	8.55
As	0.00	0.00	0.00
Cu	15.35	17.22	19.35

## Data Availability

Data for this article were generated and analyzed during the current study.
